# Overcoming resistance of targeted EGFR monotherapy by inhibition of STAT3 escape pathway in soft tissue sarcoma

**DOI:** 10.18632/oncotarget.7452

**Published:** 2016-02-17

**Authors:** Xiaochun Wang, David Goldstein, Philip J. Crowe, Mark Yang, Kerryn Garrett, Nikolajs Zeps, Jia-Lin Yang

**Affiliations:** ^1^ Department of Surgery, Adult Cancer Program, Lowy Cancer Research Centre, Clinical School of Prince of Wales Hospital, Faculty of Medicine, University of New South Wales, Sydney, Australia; ^2^ Department of Medical Oncology, Adult Cancer Program, Lowy Cancer Research Centre, Clinical School of Prince of Wales Hospital, Faculty of Medicine, University of New South Wales, Sydney, Australia; ^3^ Sarcoma and Nanooncology Group, Adult Cancer Program, Lowy Cancer Research Centre, Clinical School of Prince of Wales Hospital, Faculty of Medicine, University of New South Wales, Sydney, Australia; ^4^ Bendat Family Comprehensive Cancer Centre, St John of God HealthCare, Perth, Australia; ^5^ School of Surgery, The University of Western Australia, Perth, Australia

**Keywords:** STAT3, EGFR, sarcoma, resistance, targeted therapy

## Abstract

Although epidermal growth factor receptor (EGFR) is often over-expressed in soft tissue sarcoma (STS), a phase II trial using an EGFR inhibitor gefitinib showed a low response rate. This study identified a new secondary resistance mechanism of gefitinib in STS, and developed new strategies to improve the effectiveness of EGFR inhibition particularly by blocking the STAT3 pathway.

We demonstrated that seven STS cell lines of diverse histological origin showed resistance to gefitinib despite blockade of phosphorylated EGFR (pEGFR) and downstream signal transducers (pAKT and pERK) in PI3K/AKT and RAS/ERK pathways. Gefitinib exposure was not associated with decrease in the ratio of pSTAT3/pSTAT1. The relative STAT3 abundance and activation may be responsible for the drug resistance. We therefore hypothesized that the addition of a STAT3 inhibitor could overcome the STAT3 escape pathway.

We found that the addition of STAT3 inhibitor S3I-201 to gefitinib achieved synergistic anti-proliferative and pro-apoptotic effects in all three STS cell lines examined. This was confirmed in a fibrosarcoma xenografted mouse model, where the tumours from the combination group (418mm^3^) were significantly smaller than those from untreated (1032mm^3^) or single drug (912 and 798mm^3^) groups.

Our findings may have clinical implications for optimising EGFR-targeted therapy in STS.

## INTRODUCTION

Soft tissue sarcoma (STS) is a malignancy that arises from transformed cells of mesenchymal origin. It was estimated that about 12,020 new cases of STS were diagnosed and 4,740 patients died from STS in the USA in 2014 [1]. Current treatment for STS relies upon aggressive surgery, often in combination with radiotherapy and chemotherapy. However, approximately half of all patients will die of local recurrence or metastatic disease within 5 years [2]. Our current drug therapies have suboptimal outcomes and new treatments are needed.

Epidermal growth factor receptor (EGFR) is activated following ligand binding to its extracellular domain. This leads to phosphorylation of critical tyrosine residues which activates signalling cascades and induces gene transcription [3]. These include the RAS/RAF/MAPK (mitogen activated protein kinase)/ERK (extracellular signal-regulated kinase (ERK, MAPK1), the PI3K (phosphatidylinositol 3-kinase, PIK3CA)/AKT (Protein kinase B)/mTOR (mammalian target of rapamycin) and the JAK (Janus kinase)/STAT (signal transducers and activators of transcription) pathways. The activation of these pathways stimulates cellular proliferation, growth, survival and mobility [4].

In epithelial tumours, EGFR is often over-expressed and contributes to many cellular processes — cell cycle progression, angiogenesis, metastases and anti-apoptosis [5]. Therefore, EGFR is an important molecular target. Its inhibition (such as gefitinib as the first selective EGFR inhibitor) is now well established clinically in several epithelial-origin tumours that display functional dysregulation of this receptor [6, 7]. Those who respond often have very prolonged benefit but do not lead to cure [8]. Gefitinib selectively binds to the ATP binding pocket of the phosphorylation sites on the EGFR tyrosine kinase (TK) domain, thus blocking EGFR activation and EGFR downstream signal transduction pathways.

We and others have reported that total EGFR (tEGFR) was highly expressed in STS and significantly associated with histological grade, but was not an independent prognostic factor of survival [9, 10]. Our unpublished data indicated that phosphorylated EGFR (pEGFR) was an independent prognostic factor of survival in STS patients. These findings indicated that suppressing EGFR may greatly benefit sarcoma patients. However, a phase II clinical trial in advanced synovial sarcomas demonstrated that single agent gefitinib was unsatisfactory with low response rates and short disease control [11]. Therefore, identification of the mechanism of gefitinib resistance in STS and therapeutic combinations with both a higher proportion of responders and potentially more sustained benefit is needed.

In that light the interaction of the EGFR and JAK/STAT pathways is of interest. The STATs exert diverse actions on gene transcription and protein translation. Upon activation, STATs form homo- or hetero-dimers, translocate into the nucleus and bind to a variety of targets [12]. There are two key STAT subtypes [13]: STAT3, an oncogene which promotes cell survival and proliferation, and STAT1, a tumour suppressor which induces anti-proliferative and pro-apoptotic responses. The balance of STAT3/STAT1 regulates tumourigenesis directly by modulating STAT-dependent target genes or indirectly by controlling angiogenesis or antitumour immune responses [12]. The oncogenic effect of STAT3 has led to intensive efforts to develop STAT3 inhibitors [14]. STAT3 inhibitors have been shown to have anti-proliferative and pro-apoptotic effects *in vitro* and *in vivo* in several cancers [15, 16] and have entered clinical trials (NCI Glioblastoma Clinical Trial No. NCT00696176 and [17]). Recent studies have identified an association between EGFR and STAT3. Activated wild-type EGFR has been reported to physically associate and colocalize with STAT3 in the nucleus leading to direct transcriptional activation of the pro-oncogenic genes VEGF and iNOS in breast cancer cells [18]. STAT3 forms a complex with the oncoprotein EGFR type III variant (EGFRvIII) in the nucleus and thereby mediates EGFRvIII-induced glial transformation [19]. Given the insufficient clinical efficacy of anti-EGFR therapy in sarcoma, our aim was to investigate the mechanism of gefitinib resistance and therapeutic combinations overcoming the resistance to improve the efficacy of targeted-therapies in sarcoma.

## RESULTS

### Protein expression of EGFR and its downstream signal transducers in a panel of seven STS cell lines

**Figure 1:**
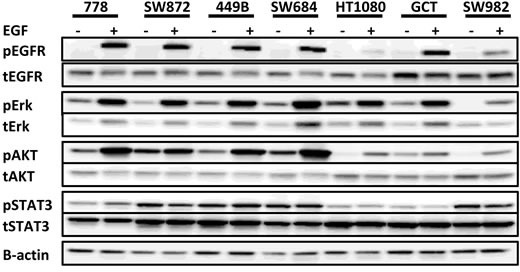
Expression of total and activated EGFR and its downstream signalling transducers in a panel of seven soft tissue sarcoma cell lines Representative images of Western blot from duplicate experiments, exhibiting pEGFR^Tyr1068^, tEGFR, pAKT^Ser473^, tAKT, pERK^Thr202/Tyr204^, tERK, pSTAT3^Tyr705^, tSTAT3.

Firstly, we analyzed the baseline levels of EGFR and phosphorylation status in the absence/presence of EGF by Western blot in seven STS cell lines representing different histotypes (Figure 1 & Table 1). All were positive in tEGFR expression at varying levels. EGF stimulation induced pEGFR in all cell lines, while pEGFR was undetectable/weak in the absence of EGF. We also examined the expression of EGFR relevant downstream signal transducers in these cell lines. It was found that pAKT and pERK were detected in the majority of cell lines in the absence of EGF and the levels were all significantly increased following EGF stimulation (mimicking closely the *in vivo* setting) (*p* < 0.05, Figure 1). The expression of pSTAT3 was at a high level in the absence of EGF. Although EGF treatment had a mild effect on upregulation of pSTAT3 levels in 778 and 449B cell lines, this did not reach statistical significance (*p >* 0.05). Immunohistochemistry studies were also used to better understand the pattern of these proteins in cells (supplementary Figure S1). Both 778 and SW872 showed very strong positive staining against total EGFR, AKT, ERK and STAT3 as well as moderate positive staining for phosphorylated proteins, except for relative weakness for 778 against pSTAT3, which is consistent with our Western blot data.

### *EGFR*, *KRAS* and *BRAF* mutations in STS cell lines

Seven STS cell lines were initially screened for mutations in the TK domain (exons 18-24) of the *EGFR* gene to rule out potential interference by the target's alteration. No rare sequence variants were detected. Single nucleotide polymorphisms in exon 20 (rs10251977, Gln787Gln G > A) and exon 23 (rs1140475, Thr903Thr C > T) occurred at an allele frequency of 0.73 and 0.09, respectively. On mutation analysis of *KRAS* and *BRAF* genes, all STS cell lines were found to be *KRAS* wild-type at codons 12, 13 and 61. SW872, SW982 and GCT (3/7 STS cell lines) demonstrated the *BRAF V600E* mutation (dbSNP:rs113488022, p.Val600Glu) (Table 1).

### Therapeutic effect of gefitinib on seven STS cell lines

The anti-proliferative effects of gefitinib on these seven STS cell lines were determined by crystal violet colorimetric assay. The mean IC_50_s were 13.17-32.82μM (Table 1), while it was 0.018μM for PC9 (human adenocarcinoma cell line), which served as positive control. Using the standard previously described in a similar lung cancer study (the sensitivity threshold of gefitinib: IC_50_≤10μM) [20], our results indicated that all STS cell lines were resistant to gefitinib mono therapy. Consistently, anti-EGFR siRNA failed to exert an anti-proliferative effect (Supplementary Figure S1).

In addition there was no significant correlation between sensitivity (IC_50_) of STS cell lines to gefitinib and EGF-stimulated pEGFR expression or tEGFR (Table 1, *p* > 0.05) and IC_50_ values of gefitinib were not statistically correlated with *BRAF* mutational status, indicating the *BRAF* mutation may not activate in the resistance to gefitinib in this panel of STS cell lines.

**Table 1:** Correlation analysis of gefitinib treatment on STS cell lines

Sarcoma subtype	Cell line	*BRAF* status	tEGFR expression	EGF-stimulated pEGFR	IC_50_ of gefitinib (μM)^a^
Liposarcoma	778	Wild type	1.63±0.29	2.37±0.20	28.29 ± 8.69
	449B	Wild type	1.28±0.26	2.03±0.55	28.95 ± 2.87
	SW872	V600E	1.99±0.19	2.98±0.29	21.60 ± 5.09
Fibrosarcoma	HT1080	Wild type	1.46±0.17	0.34±0.10	13.65 ± 4.42
	SW684	Wild type	1.39±0.47	2.56±0.52	32.82 ± 1.84
Synovial sarcoma	SW982	V600E	1.69±0.06	0.65±0.08	14.09 ± 2.86
Fibrous histiocytoma	GCT	V600E	2.00±0.26	1.82±0.04	13.17 ± 1.49
**All seven STS cell lines correlation analysis**					
IC_50_ versus tEGFR				*p* = 0.259; r = −0.529	
IC_50_ versus EGF-stimulated pEGFR				*p* = 0.102; r = 0.694	
IC_50_ of mutant *BRAF* versus IC_50_ of wild-type *BRAF*				*p* = 0.124	

tEGFR: total EGFR; pEGFR: phosphorylated EGFR

a PC9 (human adenocarcinoma cell line, served as positive control): IC_50_ = 0.018 μM

### Effect of gefitinib on the activation of EGFR and its downstream pathways

**Figure 2:**
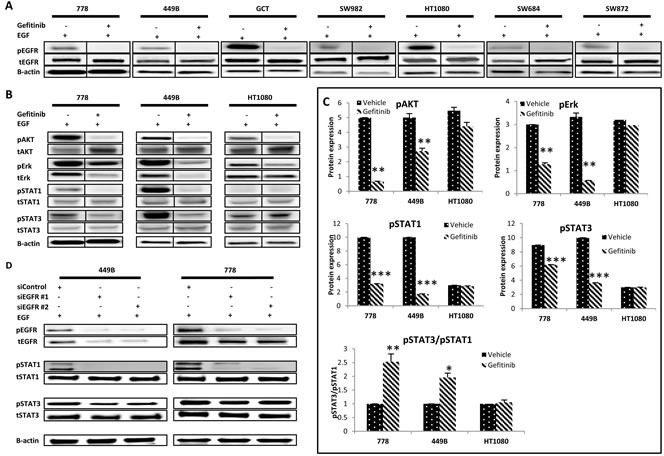
Effect of EGFR targeted monotherapy using either gefitinib or anti-EGFR siRNA on selective signalling markers in wild type STS cell lines **A.** EGFR alteration and phosphorylation followed by gefitinib treatment in a panel of seven STS cell lines. Representative images of Western blot detection of tEGFR and pEGFR ^Tyr1068^ before and after treatment with gefitinib (10 μM) for 24 hours with or without EGF stimulation. **B.** Representative images of Western blot detecting pAKT^Ser473^, pERK^Thr202/Tyr204^, pSTAT1^Tyr701^ and pSTAT3^Tyr705^ and their total proteins after treatment with vehicle control (0.08% DMSO) or gefitinib (10 μM) for 24 hours with EGF (100 ng/ml, 15 minutes). **C.** Western blot images (duplicate) were quantified using ImageQuant software. Phosphorylated proteins pAKT, pERK, pSTAT1 and pSTAT3 were normalized to the corresponding β-actin. **D.** At 48 hours post-transfection of siEGFR, cells were harvested for Western blot analysis. Point: mean of duplicate data. Error bar: standard deviation (SD). * *p* ≤ 0.05, ** *p* ≤ 0.01.

Although the EGF-induced pEGFR was completely blocked by gefitinib monotherapy in all seven STS cell lines (Figure 2A), they were all resistant to gefitinib, suggesting the existence of at least one secondary resistance mechanism.

To identify the potential escape pathways, we examined the impact of EGFR inhibition on the activity of two downstream pathways - PI3K/AKT and RAS/RAF/ERK (which were reported to be both inactivated in the gefitinib-sensitive carcinoma cell line A431 [21]), using the STS cell lines with wild-type *EGFR TK, KRAS* and *BRAF* genes (778, 449B and HT1080), so as to rule out any interference from gene mutation and using EGF to maximize downstream expression (Figure 2B and 2C, Supplementary Figure S2A). The pAKT was significantly inhibited by gefitinib treatment in the presence of EGF in 778 and 449B (% expression at gefitinib/vehicle: 778: 13%, *p <* 0.001; 449B: 55%, *p* = 0.001; HT1080: 81%, *p =* 0.09). The EGF-induced pERK was blocked (gefitinib/vehicle: 16-93%) by gefitinib, with two cell lines (778: *p* = 0.0007 and 449B: *p* = 0.0028) having statistical significance.

We then investigated both STAT1 (tumour suppressor) and STAT3 (oncoprotein) activity in JAK/STAT pathway after gefitinib monotherapy (Figure 2B and 2C). Gefitinib down-regulated both pSTAT1 and pSTAT3 in all 3 cell lines, with statistical significance in 778 and 449B (gefitinib/vehicle: pSTAT1: 32% and 17%, respectively; pSTAT3: 69% and 36%, all *p*≤0.05) but a non-significant trend for HT1080 (95% and 99%, *p* > 0.05). The ratio of pSTAT3/pSTAT1 was significantly increased in 778 (2.5 folds, *p* = 0.006) and 449B (2.0 folds, *p* = 0.025), and slightly increased in HT1080 (1.05 folds, *p* = 0.60), suggesting that gefitinib failed to decrease the ratio of oncogene pSTAT3 *versus* tumour suppressor pSTAT1. To confirm if the increased ratio of pSTAT3/pSTAT1 induced the resistance of STS cell lines to EGFR targeted therapy, we knocked down EGFR using anti-EGFR siRNA. Consistently, siEGFR downregulated much more pSTAT1 than pSTAT3 (showing increase of pSTAT3/pSTAT1) and had no anti-proliferative effect (Figure 2D, Supplementary Figure S2B and S2C).

### Gefitinib combined with S3I-201 induced synergistic anti-proliferation

**Figure 3:**
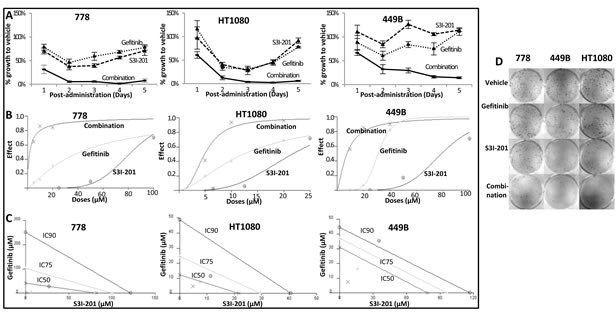
Sarcoma cell lines 778, HT1080 and 449B achieved synergistic anti-proliferative effect after combination treatment with gefitinib and S3I-201 **A.-C.** Quantitative data from crystal violet assay were analysed by CalcuSyn software. **A.** Growth curves; **B.** Dose effect curves: the effect was a measure of the relative inhibition compared to vehicle control following treatment; **C.** Isobolograms: For a given effect level, the axes represent the required combination therapy doses for IC50, IC75 and IC90. If the combination point at each IC level falls on the diagonal line, an additive effect is achieved; if it falls on the lower left of the diagonal, a synergistic effect is achieved, and if it falls on the upper right of the diagonal, an antagonistic effect is achieved. **D.** Clonogenic assay in STS cell lines treated with gefitinib (10 μM), S3I-201 (5 μM) or combination of both drugs. After colonies formation (more than 50 cells), cells were stained with crystal violet to determine the presence of colonies.

Our additional preliminary study reported that STAT3 inhibitor S3I-201 inhibited pSTAT3 in the majority of STS cell lines [22], with the IC_50_s on the three wild-type STS cell lines 778, 449B and HT1080 being 148.5, 95.5 and 14.5μM, respectively (sensitivity threshold for S3I-201: IC_50_≤100μM). In subsequent combination therapy study, these three cell lines were exposed to gefitinib, S3I-201 or gefitinib plus S3I-201 at a fixed ratio for 5 days and stained by crystal violet. The results were analysed by CalcuSyn software (UK) as shown in Table 2. Our data showed that combination treatment achieved synergistic anti-proliferation in all 3 STS cell lines (Combination Index [CI] < 1; except 449B at IC_90_), with the most synergistic effect in 778 (CI = 0.1-0.3; defined as strong synergy). For the most synergistic cell line 778, the drug reduction index (DRI; which measures how many folds the dose of each drug may be reduced when drugs were combined at the IC_50_ effect level compared with the dose of drug alone) for gefitinib and S3I-201 was 5.97 and 15.74, respectively, suggesting that the combination therapy using approximate 6-fold reduction of gefitinib (4.74μM) and 16-fold reduction of S3I-201 (9.43μM) can achieve the same anti-proliferative effect as a full IC_50_ dose of either gefitinib or S3I-201 monotherapy. The combination therapy also induced dose reduction potential in 449B (Table 2, DRI = 4.15 and 10.56) and HT1080 (2.53 and 4.45), which indicated that 6.98μM gefitinib plus 9.04μM S3I-201 in 449B or 1.44μM gefitinib plus 3.26μM S3I-201 in HT1080 could achieve IC_50_ effect. The implication of this is that S3I-201 could overcome the resistance of gefitinib monotherapy and bring the needed concentration of gefitinib in the combination therapy to achieve IC_50_ effect lower than the sensitivity threshold (10μM) in all these three examined cell lines. Figure 3 shows growth inhibition curves (A) following combination compared to monotherapy, as well as dose-effect curves (B) and isobolograms (C) produced by CalcuSyn. To better understand the synergism of the combination use of these two drugs, we explored multiple IC_50_ ratios (gefitinib:S3I-201 = 1:1, 1:2, 1:4, 2:1 and 4:1) and different sequences (concurrent, pre-treatment with gefitinib for 24 hours or pre-treatment with S3I-201 for 24 hours) in 778 cell line. As shown in Supplementary Table 1, the combination treatment produced synergism at each ratio and sequence, while it appears the combination therapy using gefitinib and S3I-201 at their equipotent or relative lower doses of gefitinib produced stronger synergism in 778 cell line, and the combination therapy worked best in parallel.

Combination therapy was also investigated by colony formation assay to observe long-term growth inhibition effect. As shown in Figure 3D and Supplementary Figure S3, synergistic anti-colony formation was consistently achieved, showing that gefitinib and S3I-201 in combination was significantly more effective than single treatments in inhibiting sarcoma cell colony-formation (all *p* < 0.05). In detail, the survival fraction was 35% (778), 21% (449B) and 41% (HT1080) for combination treatment, whereas it was 85% and 84% (778), 57% and 73% (449B), 69% and 83% (HT1080) for monotherapy.

**Table 2:** Synergistic analysis for combination therapy via CalcuSyn software

Cell line	Combination Index (CI)			Drug Reduction Index (DRI)		IC_50_ (μM)		Synergism/antagonism
	IC_50_	IC_75_	IC_90_	Gefitinib	S3I-201	Gefitinib	S3I-201	
778	0.23	0.15	0.14	5.97	15.74	4.74	9.43	Strong Synergism
449B	0.34	0.61	1.10	4.15	10.56	6.98	9.04	Synergism
HT1080	0.62	0.55	0.51	2.53	4.45	1.44	3.26	Synergism

### Combination therapy significantly decreased pSTAT3, perturbed the ratio of pSTAT3/pSTAT1 and enhanced apoptosis

**Figure 4:**
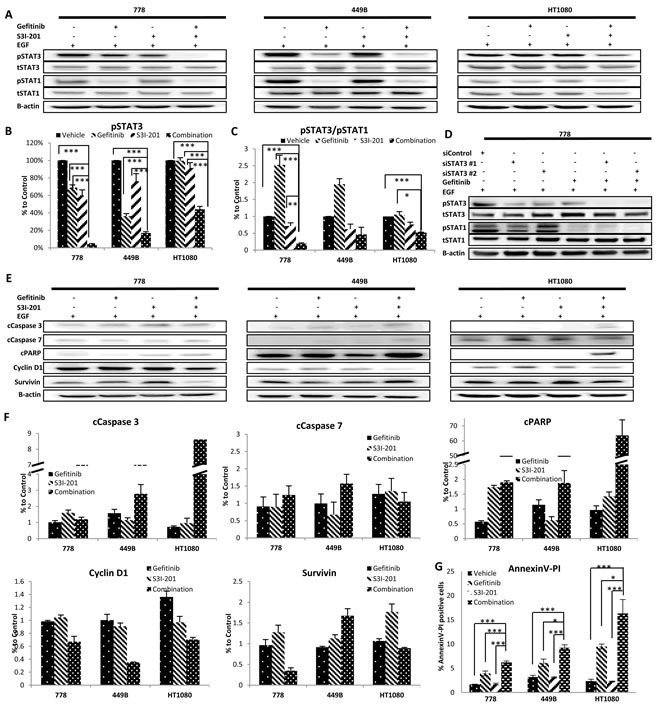
Combination therapy using gefitinib and S3I-201 induced further inhibition of pSTAT3, reduction of pSTAT3/pSAT1 and induced apoptosis and cell cycle arrest **A.** Representative images of Western blot analysis of total (t) and phosphorylated (p) STAT3 and STAT1 before and after treatment with gefitinib (10μM) and/or S3I-201 (25μM) for 24 hours in the presence of EGF on three wild type STS cell lines. **B.**-**C.** Data from at least triplicate experiments were quantified using ImageQuant software. The pSTAT3 expression in individual cell lines in the presence of EGF was normalized to β-actin and shown as percent (%) expression of post *versus* pre-treatment, as well as the ratio of pSTAT3/pSTAT1. **D.** At 24 hours post-transfection of anti-STAT3 siRNA, cells were treated with 10μM gefitinib. After incubation for 24 hours, cells were harvested for Western blot analysis. **E.** Representative imaging of Western blot of (from top row to bottom) cleaved**C.** Caspase-3, cCaspase-7 and cPARP, Cyclin D1 and Survivin before and after treatment with gefitinib and/or S3I-201 for 24 hours with EGF in 3 STS cell lines (778, 449B and HT1080). **F.** Data from at least triplicate experiments were quantified using ImageQuant software. All proteins with EGF stimulation were normalized to β-actin and were shown as percentage (%) expression of post/pre-treatment. **G.** Cells were treated with vehicle control (DMSO), gefitinib and/or S3I-201 for 24 hours and subjected to Annexin V/PI (propidium iodide) staining and flow cytometry. Error bar: standard deviation (SD). Error bar: standard deviation (SD).* *p* < 0.05, ** *p* < 0.01, *** *p* < 0.005.

To investigate the molecular mechanisms that underlie the observed synergism, STS cell lines were treated with vehicle, gefitinib (10μM), S3I-201 (25μM) or combination in the absence/presence of EGF. Whole cell lysates were used for Western blot to detect STAT3 and STAT1 (Figure 4A). Our data showed that combination therapy significantly inhibited STAT3 phosphorylation by 56-96% compared to the corresponding untreated/vehicle control (all *p* < 0.005). Although gefitinib monotherapy showed pSTAT3 inhibition to a certain extent, combination treatment together with S3I-201 induced further down-regulation in 778, 449B and HT1080 by 94%, 53% and 56%, respectively, compared to gefitinib alone (all *p* < 0.001) (Figure 4B). Importantly, the addition of S3I-201 to gefitinib dramatically perturbed the ratios of pSTAT3/pSTAT1 in all of them compared to vehicle control (combination/vehicle: HT1080: 52%, *p* = 0.0006; 449B: 47%, *p* = 0.24 and 778: 19%, *p* < 0.001) or gefitinib alone (combination/gefitinib: HT1080: 49%, *p* = 0.027; 449B: 24%, *p* = 0.15 and 778: 7.7%, *p* = 0.0036) (Figure 4C). Similarly, concurrent treatment with STAT3-specific siRNA replacing S3I-201 further blocked STAT3 activation and decreased the ratio of pSTAT3/pSTAT1 (Figure 4D) supporting the specificity of S3I-201.

We further examined the alteration of protein markers regulating apoptosis (Figure 4E and 4F) by Western Blot and found that combination therapy strongly increased the expression of apoptotic markers including cleaved caspase (cCaspase)-3 (449B: 1.8 times; HT1080: 11.8 times), cCaspase-7 (778: 1.4 times; 449B: 1.58 times) and cleaved Poly ADP-Ribose Polymerase (cPARP) (778: 3.34 times; 449B: 1.65 times; HT1080: 65.93 times) compared to gefitinib monotherapy. We also examined the effect on survivin (a member of the inhibitor of apoptosis family) and cyclin D1 (cell cycle regulator). It was found that cyclin D1 was decreased after combination treatment compared to gefitinib alone in all three cell lines (combination/gefitinib: 449B: 35%; 778: 68%; HT1080: 52%) and survivin was also downregulated in 778 (36%) and HT1080 (84%) but not in 449B (183%). To confirm apoptotic effect by combination therapy, we further detected apoptosis using Annexin V and propidium iodide (PI) binding by flow cytometry. Figure 4G shows the combination therapy significantly enhanced apoptosis compared to the vehicle control and single drug treatments in wild-type STS cell lines.

### *In vivo* supra-additive antitumour growth effect induced by concurrent usage of gefitinib and S3I-201 in human fibrosarcoma xenografted nude mouse model

**Figure 5:**
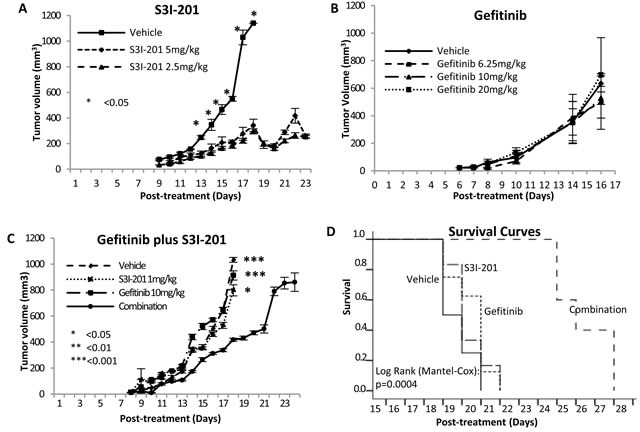
Combination therapy supra-additively enhanced the fibrosarcoma growth inhibition and delay in mouse model **A.** S3I-201 induced significant tumour growth inhibition and delay in orthotopic fibrosarcoma xenografted nude mice. 0.1 × 10^6^ HT1080 cells were injected intramuscularly into the right posterior thigh musculature of each nude mouse. On 24 hours post inoculation of tumour cells, all mice started treatment with vehicle, 2.5 or 5 mg/kg S3I-201 *via* intraperitoneal injection once daily. *N* = 3-4 **B.** Gefitinib (up to 20 mg/kg) did not induce tumour growth inhibition in orthotopic fibrosarcoma xenografted nude mice. *N* = 5 **C.** Combination therapy synergistically enhanced the fibrosarcoma growth inhibition. *N* = 8 **D.** Kaplan-Meier survival curve of human fibrosarcoma xenografted mice comparing single or combination therapy. The difference was significant (log-rank test). *N* = 8.

To extend the investigation to *in vivo*, a human fibrosarcoma HT1080 xenografted nude mouse model was identified as the optimized choice among above 3 wild-type STS cell lines, considering that neither 778 was tumorigenic in balb/c nude mice (our unpublished data) nor 449B in NOD SCID mice [23]. Prior to combination therapy, we tested two small preliminary studies with single drug S3I-201 or gefitinib. After 24 hours intramuscular inoculation of HT1080, mice were randomly divided and treated by vehicle DMSO, 2.5mg/kg S3I-201 or 5mg/kg S3I-201, which was given intraperitoneally. On day 9-10 after treatment, tumours of about 4-5mm in diameter were formed in all groups. From day 13 post-treatment, a significant inhibition of tumour growth was found in S3I-201-treated groups at either dose (*p* < 0.05, Figure 5A) compared to vehicle control group. On Day 18, tumours from vehicle control group reached approximately 15mm in diameter and 1140mm^3^ in volume, while tumours from treatment groups were approximately 382 and 295mm^3^ (Vehicle/S3I-201 *p* < 0.005). Consistently with *in vitro*, as shown in Figure 5B, there was no anti-sarcoma effect of gefitinib *in vivo* even with doses of up to 20mg/kg (*p* > 0.05).

We next examined whether using S3I-201 could also improve the effectiveness of gefitinib using our mouse model in two independent combination therapy experiments. Twenty-four hours post-inoculation of HT1080, all mice were randomly divided into 4 groups and treated with vehicle control (1% Tween 80), 1mg/kg S3I-201 (intraperitoneally), 10mg/kg gefitinib (gavage) or combination with 1mg/kg S3I-201 and 10mg/kg gefitinib once daily. These two doses were specifically selected so that their independent effect on tumour growth inhibition would be modest and the concentrations were clinically achievable [24-26]. The combination therapy significantly enhanced the inhibition and delay of tumour growth compared to vehicle control and monotherapy. On day 18 post-treatment in the first experiment, the tumours from combination therapy group (418mm^3^) were significantly smaller than those from untreated (1032mm^3^) and single drug treated (912 and 798mm^3^) groups (non-parametric and parametric methods: combination/vehicle *p* < 0.001; combination/gefitinib monotherapy *p* < 0.001; combination/S3I-201 *p* < 0.05) (Figure 5C). All mice in vehicle and monotherapy groups were sacrificed on day 18 because at least one tumour in its group reached about 1000mm^3^, whilst all mice treated by combination were continually treated with both drugs for a further six days. To show the survival effect of combination therapy, in the second experiment, each individual mouse was sacrificed once its tumour reached about 1000mm^3^. Kaplan-Meier survival curves (Figure 5D) shows that combination therapy had significantly prolonged benefit (mean of survival time [days]: vehicle: 18.8, gefitinib: 19.5, S3I-201: 19.3 and combination: 25.4; *p* = 0.0004). Importantly, all treated mice tolerated S3I-201 and gefitinib well, showing general good health with no signs of distress. All groups showed no body weight loss more than 20% and there was no significant difference in body weight changes between untreated and treated groups (*p* > 0.05) (Supplementary Figure S4). For drug toxicity assessment, all tumours and organs (lung, heart, liver, spleen and kidney) were harvested at the end points and showed no macromorphological and histological abnormalities (Supplementary Figure S5). To further confirm whether toxicity was induced followed by the mono- or combination therapy, the blood was collected after sacrificing the mice to examine liver and kidney functions. As shown in supplementary Figure S6, all four serum biomarkers did not show significant changes after mono- or combination therapy (*p* > 0.05), supporting that the use of S3I-201 and gefitinib alone or in combination was safe and well-tolerated by nude mice.

## DISCUSSION

Soft tissue sarcomas are rare, heterogeneous mesenchymal neoplasms and most common chemotherapeutic agents offer limited benefit [27]. Despite multimodality treatment, overall sarcoma survival rates remain unsatisfactory. Recently, the therapeutic focus has been on targeting the biological mechanisms driving tumourigenesis. Our unpublished studies on sarcoma patients suggested that pEGFR was independently associated with worse cancer specific survival. The over-expression of pEGFR, pAKT, pERK and pSTAT3 were found in sarcoma patients unrelated to histology, indicating EGFR-targeted therapy may benefit sarcoma patients.

We report here, in the presence of EGF ligand, which mimics closely the *in vivo* setting, pEGFR along with its signal transducers (pAKT, pERK and pSTAT3) [representing three downstream pathways] were highly expressed in the majority of STS cell lines. Furthermore, the observations that EGF stimulation significantly increased protein expression of pAKT and pERK, but not pSTAT3, indicated that both AKT and ERK pathways in sarcomas may be mainly stimulated by EGF/EGFR activation. Therefore, blocking EGFR pathway may be a good treatment approach in STS, due to the over-expression of activated EGFR and its activated downstream signal transducers.

Use of an EGFR tyrosine kinase inhibitor (TKI) is now a part of standard care in biologically appropriate subsets in cancers of the lung [7], colon [28] and head and neck [3]. In this study, we demonstrated gefitinib or anti-EGFR siRNA alone failed to exert an anti-proliferative effect in all seven STS cell lines across different histopathological subtypes, despite completely inhibiting EGFR activation. This together with the limited activity in a clinical trial using gefitinib in sarcoma [11] encouraged us to explore the potential resistance mechanisms. The sensitivity to EGFR TKIs has been positively associated with activating mutations in *EGFR* gene such as deletion mutations occurring around codon 746-750 in exon 19 and the substitution of leucine with arginine at codon 858 in exon 21 (L858) [29, 30]. In NSCLC, about 70-80% patients with activating mutant *EGFR* gene were sensitive to EGFR TKI, compared with only 10-20% response rate in wild-type *EGFR* gene [31]. A poor response to EGFR TKIs was also associated with EGFR-resistant mutations such as exon 20 insertions or *KRAS* and *BRAF* mutations [32-34]. In our mutation analysis, the lack of *EGFR TK* activating mutation in our panel of STS cell lines indicated that these cells may not be sensitive to EGFR-targeted therapy. Our result is consistent with clinical reports. In a cohort of 958 patients, only 2 of 38 samples from the sarcoma subset were positive for *EGFR* mutation [35]. In a synovial sarcoma study, only 2 of 13 tissue samples were positive for *EGFR TK* mutation, with no *EGFR* amplification on FISH analysis [36] and a further study on *EGFR* gene amplification from patients with endometrial stromal sarcoma also showed 10/10 negative results [37]. Additionally, gene mutations activating the EGFR downstream signalling pathways may mediate the primary and required resistance to EGFR-targeted therapy. It has been reported recently that *KRAS* and *BRAF* mutations were negatively correlated with the response to targeting EGFR treatment in lung and colorectal cancers [38, 39]. The *KRAS* mutation was not detected in our panel and was consistent with previous studies [40] which showed only 2 of 54 samples from patients with STS had *KRAS* mutations. Similarly, a recent study found that none of the samples from 108 sarcoma patients were *BRAF* mutation positive [41]. Unexpectedly, we discovered that 3 STS cell lines SW872, SW982 and GCT contained a *BRAF V600E* mutation. We therefore chose to focus on *BRAF* wild type cell lines as likely more representative of the human situation. Other mutations (such as non-T790M *EGFR* mutations *D761Y, L747S* and *T854A*, amplification of *c-Met*, loss of PTEN, *PIK3CA* mutations and *BIM BH3* deletion) have been reported to result in the resistance to EGFR TKIs in some cancers [42]. However, the roles of these mutations in sarcoma still remain unknown. Similarly the tumour microenvironment is an increasing area of focus in many tumour types but its role in resistance in soft tissue sarcomas is not yet well studied.

An alternative explanation for the limited therapeutic effect of gefitinib may be the existence of a survival compensatory downstream pathway – JAK/STAT escape pathway. The over-expression of pSTAT3 may implicate pSTAT3 signalling as a potential escape pathway against EGFR blockade [13]. The blockade of RAS/RAF/ERK (pERK) and PI3K/AKT (pAKT) pathways has been associated with gefitinib sensitivity in other cancer cell lines and xenografts, such as NSCLC cell lines A549, H460, PC9, A549 and QG56, which carry intrinsically active ERK or AKT pathways [21, 43]. In the current study, gefitinib treatment in our panel of STS cell lines with wild-type *EGFR TK*, KRAS and BRAF genes (so as to rule out any interference from relevant gene mutation) did not inhibit proliferation despite complete inactivation of EGF-induced EGFR phosphorylation, as well as inhibition of representative signal transducers (pAKT and pERK) of two main downstream pathways - RAS/RAF/ERK MAPK and PI3K/AKT. In addition, we also found that gefitinib did not effectively suppress the oncogenic member (STAT3) of JAK/STAT pathway, resulting in failure to decrease the ratio of pSTAT3/pSTAT1. JAK/STAT is the third EGFR downstream signalling pathway. STAT protein activation, dimerization and nuclear translocation are induced after phosphorylation of JAK by growth factors, cytokine receptors or non-receptor tyrosine kinases signalling [13]. Therefore, JAK/STAT pathway may be stimulated by HER-dependent signalling (EGFR-depedent or HER2-dependent) or HER-independent signalling (IL-6/gp130 or Src). Gefitinib as an EGFR specific inhibitor may abrogate EGFR-dependant JAK/STAT activation, but not other sources of stimulation. The incomplete inhibition of JAK/STAT pathway may contribute to the lack of response by STS cell lines to gefitinib. There are seven members in STAT family; in particular, STAT1 and STAT3 play important roles in cancer cells [13]. STAT3 promotes cancer proliferation and/or survival directly by regulating target genes of tumour cells such as survivin, cyclin D1 and Bcl-xL [13]. On the other hand, STAT1 exerts pro-apoptotic functions [12, 44]. Based on these mostly opposite functions and their complex cross-talking, we suggest that the ratio of pSTAT3/pSTAT1 may be a better indicator of the comprehensive impact of JAK/STAT signalling on regulating sarcoma cell growth than any individual analysis of pSTAT3 or pSTAT1. This comprehensive look at the biological balance between individual STAT members has been reported in other cancer situations [12, 45]. The imbalance in STAT3/STAT1 favoured oncogenesis, and appeared to direct tumourigenesis in the EGFR pathway [12, 45]. Our study suggested that the increased/unchanged ratio of pSTAT3/pSTAT1 from the JAK/STAT signalling pathway appeared to contribute to the resistance of gefitinib in STS cell lines.

Despite its accepted use in NSCLC, gefitinib has limitations as a single agent. One approach to improve EGFR-targeted therapy in cancers was combination with chemotherapy and radiation with variable success [3, 46-48]. In addition, due to complex cross-talk between signalling pathways, examining the combination of EGFR-targeted therapy with inhibition of other receptors (HER2 and IGF-1R) or EGFR downstream signal transducers (such as RAS, MAPK, AKT and MTOR) is an alternative strategy [46]. Given the multiple non-specific genetic abnormalities that characterise sarcomas, complex cross-talking between these abnormal signalling pathways may contribute to resistance. We have demonstrated that the addition of the STAT3 inhibitor to gefitinib resulted in synergistic anti-proliferation and anti-colony formation in all three STS cell lines examined (778, 449B and HT1080). We specifically selected them considering that they have wild-type *EGFR TK, KRAS* and *BRAF* genes, so as to rule out any interference from relevant gene mutation. The STAT3 inhibitor S31-201 selectively blocked STAT3 activity *via* inhibition of STAT3 dimerization and SH2 domain-mediated interference of DNA binding and transcriptional activity, while S3I-201 has minimal effect on STAT1. Western blot data showed that the combination of gefitinib and S3I-201 or gefitinib plus anti-STAT3 siRNA induced significant further down-regulation of STAT3 phosphorylation and led to substantial decrease in the ratio of pSTAT3/pSTAT1. STAT family includes many members with variable biological effect. For example STAT3 is an oncoprotein in sarcoma, whilst STAT1 is a tumour suppressor. Therefore, checking one member's activity cannot reflect the balance status between oncoproteins and tumour suppressors. This explains why the phosphorylation status of STAT3 did not show a correlation with the effectiveness to this combination therapy. The effectiveness to the combination relates more to: 1) how much the ratio (pSTAT3/pSTAT1) was increased by gefitinib monotherapy, as well as 2) the sensitivity to STAT3 inhibitor. Several studies in other solid cancers (lung and ovarian cancers) have also indicated that STAT3 activation was associated with EGFR resistance and blocking both EGFR and JAK/STAT signalling pathways at different levels (JAK inhibitors AZD1480 or P6, or siRNA against JAK or STAT3) have shown synergistic therapeutic effects compared with EGFR inhibition alone [49, 50]. We are the first to show the synergism using a STAT3 inhibitor (S3I-201) and an EGFR inhibitor (gefitinib) together in sarcoma. Although we and others [49, 50] reported that siRNA-mediated knockdown of STAT3 enhanced gefitinib sensitivity, other STAT3 inhibitors including targeting SH2, DNA binding or N-terminal domains need to be further investigated. In order to make our studies clinically relevant, we chose the gefitinib doses of 6.25, 10 and 20 mg/kg for our *in vivo* studies, as these achieve similar therapeutic levels to those seen clinically [24-26]. As expected gefitinib monotherapy had no inhibitory effect on the fibrosarcoma xenografts in this animal model. In contrast we showed a supra-additive inhibitory effect on tumour growth and prolonged survival benefit from the drug combination (S3I-201 plus gefitinib), which prolonged survival for the combination group.

In summary, we demonstrated that all STS cell lines examined in the study showed resistance to gefitinib despite blockade of pEGFR and downstream signal transducers (pAKT and pERK) in PI3K/AKT and RAS/ERK pathways. Gefitinib exposure was not associated with decrease in the ratio of pSTAT3/pSTAT1. The relative STAT3 abundance and activation may be responsible for the drug resistance. The addition of STAT3 inhibitor S3I-201 to gefitinib achieved synergistic anti-proliferation and pro-apoptotic effects in all three wild-type STS cell lines and this is confirmed in a fibrosarcoma xenografted mouse model, where the tumours from the combination group were significantly smaller than those from untreated or single drug groups.

The present study is the first in STS field to have identified STAT3 signalling, in particular pSTA3/pSTAT1 as an escape mechanism for gefitinib monotherapy and applied combination therapy to overcome the escape signalling. These discoveries provide a positive signal for proceeding to clinical trials using this combination in sarcomas, which is currently a patient group with poor outcomes and limited systemic therapy options.

## MATERIALS AND METHODS

### Cell lines and cell culture

Five human STS cell lines (HT1080, SW684, SW872, SW982 and GCT) were purchased from the American Type of Cell Culture (Manassas, Virginia, USA). Two human liposarcoma (449B and 778) were kindly provided by Professor David Thomas (Peter MacCallum Cancer Centre, Australia) and Dr Florence Pedeatour (Nice University Hospital, France). Human adenocarcinoma cell line PC9 was purchased from European Collection of Cell Cultures (Wiltshire, UK). All cells were grown in RPMI-1640, supplemented with 10% fetal bovine serum (FBS), 2mM L-glutamine and antibiotics (50 units/ml penicillin and 50μg/ml streptomycin) at 37°C in a humidified 5% CO_2_ and 95% atmosphere. These cell lines were all identified to be mycoplasma free and cell lineages were validated using short tandem repeat profiling by CellBank Australia.

### Western blot

Cells were harvested after 24 hours treatment, and total proteins were extracted and measured using Western blot with our standard procedures [22] and as described in the Supplementary Materials and Methods - Western blot. EGFR inhibitor gefitinib and STAT3 inhibitor S3I-201 (NSC 74859) were purchased from Euroasian chemicals (India) and Merck (Germany), respectively.

### Mutation analysis

DNA was extracted from all sarcoma cell lines using the Qiagen kit (Qiagen, Hilden, Germany) for *KRAS* and *BRAF* mutations using bidirectional Sanger sequencing, as described in the Supplementary Materials and Methods - EGFR mutation analysis, K-ras and b-raf mutation analysis.

### Crystal violet colorimetric assay (cell proliferation assay)

Briefly, 24 hours after cells were seeded in 96-well plates, vehicle or drugs (gefitinib: 5-40 μM; S3I-201: 6.25-100 μM) were added into relevant wells. After required time period (1-5 days post-treatment), cells were washed with DPBS, stained with 0.5% crystal violet and incubated with Elution solution (0.1M Sodium citrate + 100% ethanol) for 30 minutes, followed by light absorbance at 540nm on a plate reader (Tecan; Austria).

### Clonogenic survival assay for adherent cells

Optimal numbers of single-cell suspensions were seeded in duplicate into six-well plates. After 24 hours, cells were treated with vehicle or drugs at required concentrations (gefitinib: 10μM, S3I-201: 5μM) and incubated at 37°C in a humidified 5% CO_2_ and 95% atmosphere. Once colony-formation (1 colony ≥ 50 cells) was observed, cells were washed with DPBS and stained with 0.5% crystal violet for 10 minutes at room temperature. Samples were imaged using a Molecular Imager Gel Doc XR System and analysed by QuantityOne software (Bio-rad, USA).

PE (Plating efficiency) (%) = (colonies observed)/(number of cells plated) x 100

Survival fraction = colonies from drug-treated cells/colonies from untreated cells.

### Transfection of siRNA

An optimal siRNA (Qiagen) concentration (25 nM for siEGFR and 10 nM for siSTAT3) in HiPerFect Transfection reagent (Qiagen) was used for transfection according to the manufacturer's instructions. 48 hours post-trasfection, cells were processed for Western blot or proliferation assay.

### Combination therapy

Based on Chou and Talalay method for combination therapy [51], four groups (vehicle, gefitinib, S3I-201, and gefitinib plus S3I-201) were used for at least duplicate independent experiments with multiple drug doses (gefitinib: 5-40 μM; S3I-201: 6.25-100 μM) and triplicate samples. The combination therapy was designed with “constant ratio two drug combination”, using single drug treatment IC_50_ results obtained from the monotherapy to guide experiment design and data were analysed using CalcuSyn software (UK). An automatically computed combination index (CI) determined at 50%, 75% and 90% inhibition of cell growth was derived based on both the potency (IC_50_) and shape of the software-generated dose-effect curves. CI < 1, = 1 and > 1 indicates synergistic, additive and antagonistic effects, respectively.

### Animal experiments

All animal experiments were approved by UNSW Animal Care and Ethics Committee. Five-week Balb/c nude mice were obtained from the Animal Resources Centre (Perth, Australia). Based on our optimisation test, 0.1 × 10^6^ HT1080/mouse were intramuscularly injected into the right back leg. After 24 hours, mice were randomly divided and treated daily by vehicle, S3I-201 (intraperitoneally), gefitinib (gavage) or S3I-201 plus gefitinib. After our two small preliminary studies for monotherapy with S3I-201 or gefitinib, we performed two independent combination therapy experiments with two different end-points: 1) all mice in the same group were sacrificed when at least one tumor volume in its group reached about 1000mm^3^ and 2) individual mouse was sacrificed once its tumor reached about 1000mm^3^. Mice were monitored daily as described in the Supplementary Materials and Methods - Animal experiments.

### Statistical analysis

Quantitative data are presented as means ± standard deviation (SD) of all replicates. The correlation between IC_50_ of gefitinib and protein expression and *BRAF* mutation status were examined by Pearson's correlation coefficient methods. The difference between matched or independent groups was analysed using paired or unpaired Student's *t*-test. Protein expression or colony formation in different treatment groups was analysed using ANOVA first. Significant ANOVA group was further analysed by a post-hoc Bonferroni test. The difference of tumour volumes between groups was analysed using both non-parametric (Kruskal-Wallies test and Tamhane Post Hoc Test) and parametric methods (oneway ANOVA and Bonferroni Post Hoc Test). Statistical analysis was performed using IBM Statistics 22 (IBM SPSS, USA). The *p*-values (2-tailed) of < 0.05 were considered statistically significant.

## SUPPLEMENTARY TABLE AND FIGURES


