# Supplementary material for: Overcoming resistance of targeted EGFR monotherapy by inhibition of STAT3 escape pathway in soft tissue sarcoma
Source: Oncotarget. 2016 Feb 17;7(16):21496–509. doi: 10.18632/oncotarget.7452 (PMC5008301; doi:10.18632/oncotarget.7452)
Supplement: Supplementary file 1 [file oncotarget-07-21496-s001.pdf]

Overcoming resistance of targeted EGFR monotherapy by inhibition of STAT3 escape pathway in soft tissue sarcoma

Supplementary Materials and Methods:

Western blot

Briefly, cells were starved overnight in RPMI containing 1% FBS and then treated with vehicle DMSO (0.08%) or drugs (20µM of gefitinib and/or 25µM of S3I-201) for 24 hours. Cells were then harvested after 15 minutes incubation with or without 100 ng/ml EGF (Sigma-Aldrich, USA) stimulation, and total proteins were extracted using RIPA buffer (Sigma-Aldrich, USA) with 1% Protease and Phosphatase inhibitor cocktails (#539131 Merck Germany and #78420 Thermofisher Australia). Protein concentrations were determined by BCA protein assay (Thermofisher), according to the company instructions. Subsequently, proteins were separated by 4-20% SDS-PAGE and transferred onto nitrocellulose membranes (Bio-rad, Australia), which were then probed with primary antibodies overnight at 4 ^{o}C. HRP-conjugated secondary antibodies were detected by chemiluminescence agent Supersignal Western Dura Extended Duration (Thermofisher, Australia). Membranes were imaged by ImageQuant LAS4000 (GE healthcare, Sweden). Densitometric analysis was performed by ImageQuant TL Software (GE healthcare) and presented as ratios of protein expression normalized to relevant β-actin loading control. All antibodies used were purchased from Cell Signaling Technology (Danvers, MA).

EGFR mutation analysis

DNA was extracted from all sarcoma cell lines using the Qiagen kit (Qiagen, Hilden, Germany) and all samples were sent to Vanessa Hayes (Cancer Research Program, Garvan Institute of Medical Research, Sydney). The entire tyrosine kinase domain of the *EGFR* gene, including exons 18 through to 24 and all relevant donor/acceptor splice sites, was amplified

by polymerase chain reaction (PCR, primer sequences are shown below) and pre-screened for sequence variation using denaturing gradient gel electrophoresis (DGGE) as outlined in Vermeij 2008 [1]. Amplicons showing aberrant DGGE banding patterns were sequenced (ABI 3100 sequencer, using dye terminator sequencing kit of Applied Biosystems (Foster City)).

EGFR primers for denaturing gradient gel electrophoresis

Gene/Exon		First Step PCR	Second Step PCR
EGFR Exon 18	F[a]	AGCATGGTGAGGGCTGAG	[C]GCTGAGGTGACCCTTTGTCTC
	R[b]	ACAGCTTGCAAGGACTCTGG	ACAGCTTGCAAGGACTCTGG
EGFR Exon 19	F	CATGTGGCACCATCTCACA	CATGTGGCACCATCTCACA
	R	CCACACAGCAAAGCAGAAAC	[C]GGTGTGTCGTTTCGTCTTTG
EGFR Exon 20	F	CGAAGCCACACTGACGTG	CGAAGCCACACTGACGTG
	R	CTATCCCAGGAGCGCAGAC	CCCGTATCTCCCTTCCCTGAT
EGFR Exon 21	F	CCTCACAGCAGGGTCTTCTC	CCTCACAGCAGGGTCTTCTC
	R	AATGCTGGCTGACCTAAAGC	[C]CCGACTGGATTTCG
EGFR Exon 22	F	TTTTTCCAACAGAGGGAAAC T	[C]CACTGCCTCATCTCTCACCA
	R	AAAGAAAATACTTGCATGTC AGAGG	AAAGAAAATACTTGCATGTCAG AGG
EGFR Exon 23	F	CCACTGCCTTCTTTTCTTGC	[C]TTTCTTGCTTCATCCTCTCAG
	R	CAGCTAGGCAGTGTGGACAG	CAGCTAGGCAGTGTGGACAG
EGFR Exon 24	F	GCATCACCAATGCCTTCTTT	[C]GCAATGCCATCTTTATCATTTC
	R	ACTCTTCCCAATGGAAGCAC	ACTCTTCCCAATGGAAGCAC

EGFR primers used two steps with a hemi-nested PCRs approach.

[a]: F=forward; [b]: R=reverse;

[c]: indicates that a GC clamp was added at the 5' end of the primer, the GC clamp = cgcccgccgcgccccgcgcccggcccgccgcccccgcccg

K-ras and b-raf mutation analysis

DNA samples from sarcoma cell lines was sent to Kerryn Garrett and Nikolajs Zeps (Bendat Family Comprehensive Cancer Centre, St John of God Health Care, Perth, Australia and School of Surgery, The University of Western Australia, Perth, Australia) for k-ras and b-raf

mutations using bidirectional Sanger sequencing. Amplicons of k-ras exons 2 and 3 covering codons 12 and 13, and 61 respectively, as well as exon 15 of b-raf covering codon 600 were amplified and the resulting PCR products sequenced (ABI 3100 sequencer, using big dye terminator sequencing kit from Applied Biosystems (Foster City]). The PCR primer sequences are shown below.

K-ras and *b-raf* primers for DNA sequencing

Gene/Exon		PCR amplification primers (5' – 3')
K-ras Exon 2	F[a]	TGTAAAACGACGGCCAGTTGTGACATGTTCTAATATAGTCACAT
	R[b]	CAGGAAACAGCTATGACCACCAGTAATATGCATATTAAAACAAGA
K-ras Exon 3	F	TGTAAAACGACGGCCAGTGACTGTGTTTCTCCCTTCTCA
	R	CAGGAAACAGCTATGACCAGCTTATTATATTCAATTTAAACCCAC
B-raf Exon 15	F	TGTAAAACGACGGCCAGTCTAAACTCTTCATAATGCTTGCTC
	R	CAGGAAACAGCTATGACCTCTAGTAACTCAGCAGCATCTCA
[C]M13 sequencing primer	F	TGTAAAACGACGGCCAGT
	R	CAGGAAACAGCTATGACC

[a]: F=forward; [b]: R=reverse;

[c]: Forward primers have M13 F sequence attached at the 5' end and reverse primers have M13 R sequence at the 5' end to help facilitate the sequencing process with a common sequencing F and R primer.

Immunohistochemistry

Cells were cultured in 8-well chamber slides at optimal density and allowed to settle overnight. At the end point, the growth medium was removed and each chamber was washed with DPBS and fixed with 10% neutral buffered formalin (NBF). Chamber slides then underwent antigen unmasking using citrate or EDTA according to the recommendation on the antibodies data sheet prior to blocking and incubating with primary antibodies for overnight at 4 ^{o}C. After washing 3 times, the slides were incubated with horseradish peroxidase (HRP) conjugated second antibodies, followed by incubation with DAKO liquid DAB+ chromogen

and counterstaining with Harris's haematoxylin. Negative controls were treated identically but incubated in control antibody (non-specific Ig) or the primary antibody was omitted.

Animal experiments

Tumour progression was documented daily by measurements using electronic callipers in two dimensions (d1 and d2) and the volume (V) was calculated by the standard formula for an ellipse: $V = \frac{1}{6}\pi\ (d1*d2)^{3/2}$. Mice were monitored daily for any loss of condition and were euthanized if they were found in distress, the tumours measured above 1000 mm^3 or their body weight losses more than 20%. After mice were euthanized at the end point, blood was collected and serum was sent to South Eastern Area Laboratory Services (SEALS) for toxicity test by examining the liver function (Alanine Amino Transferase, ALT and Alkaline Phosphatase, ALP) and kidney function (urea, creatinine).

References:

1. Vermeij J, Teugels E, Bourgain C, Xiangming J, in 't Veld P, Ghislain V, Neyns B and De Greve J. Genomic activation of the EGFR and HER2-neu genes in a significant proportion of invasive epithelial ovarian cancers. BMC Cancer. 2008; 8:3.

Supplementary Table 1: Synergistic interaction between Gefitinib and S3I-201 in variety of IC_{50} ratios and sequences on the viability of 778 cells

**Sequence**	**Gefitinib: S3I-201 (IC_{50} ratio)**	**Combination Index (CI)**			**Drug Reduction Index (DRI)**		**Synergism/ antagonism**
		IC_{50}	IC_{75}	IC_{90}	Gefitinib	S3I-201	
**Concurrent**	**1:1**	0.23	0.15	0.14	5.97	15.74	Strong Synergism
	**1:2**	0.19	0.17	0.15	6.20	37.43	Strong Synergism
	**1:4**	0.22	0.22	0.22	5.89	18.38	Strong Synergism
	**2:1**	0.40	0.39	0.39	2.61	58.0	Synergism
	**4:1**	0.49	0.49	0.50	2.11	83.5	Synergism
**Pre-gefitinib**	**1:1**	0.76	0.85	0.96	1.40	22.67	Synergism
**Pre-S3I-201**	**1:1**	0.37	0.40	0.45	3.80	9.40	Synergism

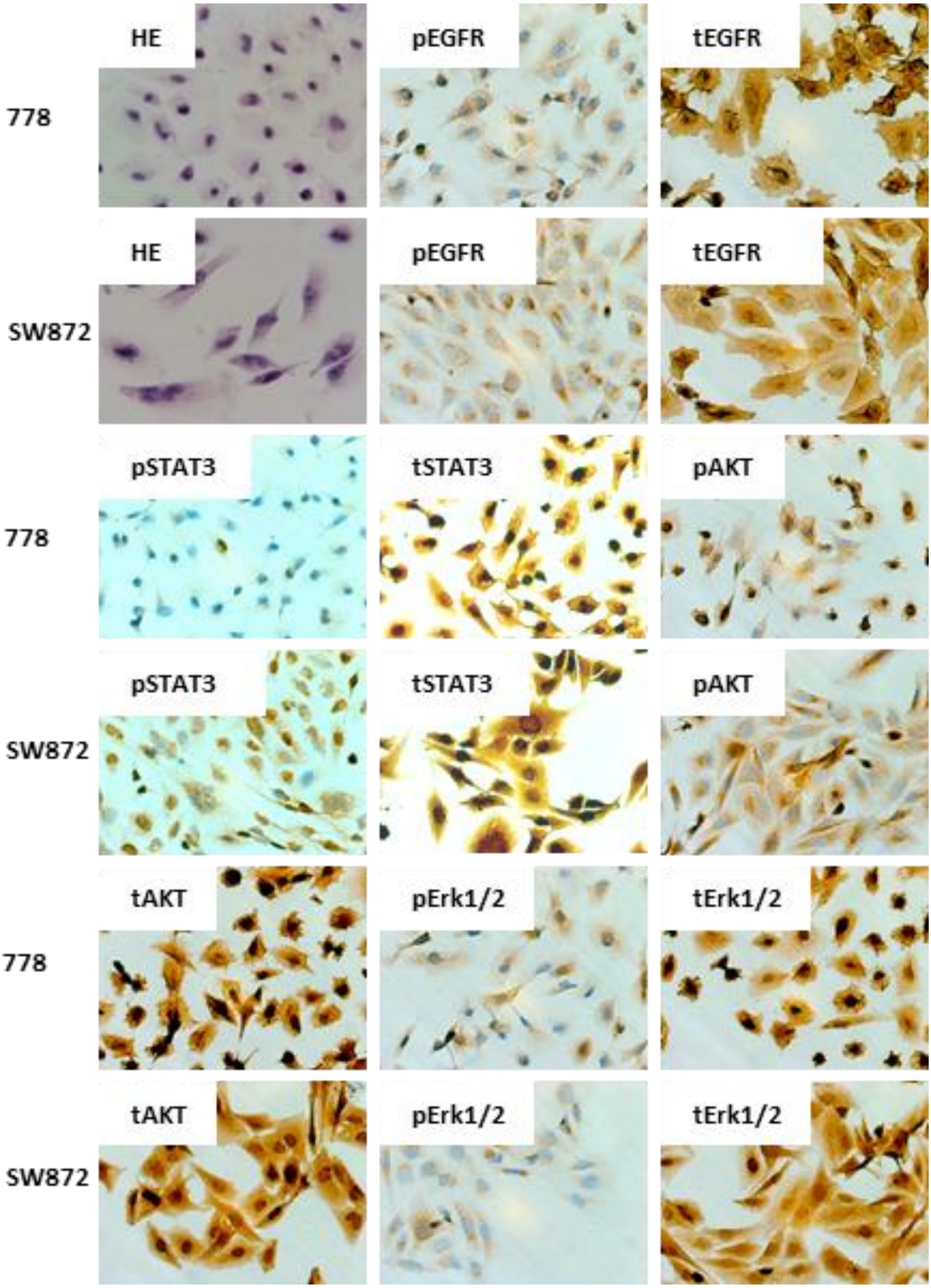

Supplementary Figure S1: Immunohistochemistry detection of EGFR, STAT3, AKT and ERK in 778 and SW872 cell lines. Cells were cultured in 8-well chamber slides at

optimal density and allowed to settle overnight. At the end point, the cells were processed by fixing, antigen unmasking, blocking and incubating with primary antibodies for overnight at 4 ^{o}C. After incubation with horseradish peroxidase (HRP) conjugated second antibodies, cells were detected with DAKO liquid DAB+ chromogen and Harris's haematoxylin as counterstaining.

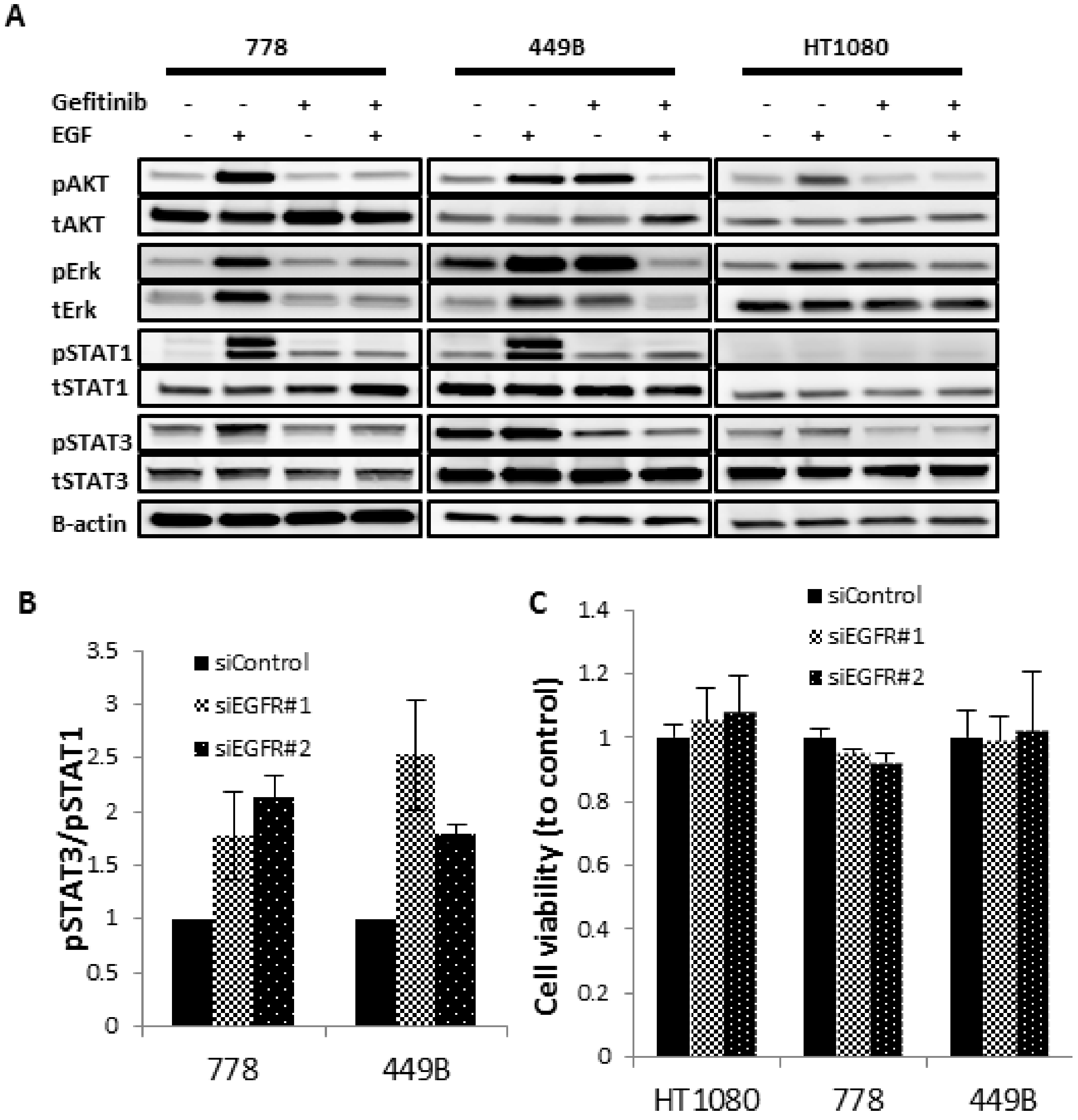

Supplementary Figure S2: Effect of EGFR targeted monotherapy using either gefitinib or anti-EGFR siRNA on selective signalling markers in wild type STS cell lines. (A) Representative images of Western blot detecting $pAKT^{Ser473}$, $pERK^{Thr202/Tyr204}$, $pSTAT1^{Tyr701}$* and $pSTAT3^{Tyr705}$ and their total proteins after treatment with vehicle control (0.08% DMSO) or gefitinib (20 µM) for 24 hours with and without EGF (100 ng/ml, 15 minutes). (B) Western blot images (duplicate) of siEGFR know-down (Figure 2D) were quantified using ImageQuant software. **(C)** Anti-EGFR siRNA failed to exert an anti-proliferative effect in HT1080, 778 and 449B. After 48 hours post-transfection of siEGFR (Qiagen SI02660140 & SI02660147), cell viability was measured using crystal violet colorimetric assay.

(*:$pSTAT1^{Tyr701}$ antibody detects endogenous levels of STAT1 only when phosphorylated at Tyr701. STAT1 protein exists as a pair of isoforms, STAT1α (91 kDa) and the splice variant STAT1β (84 kDa). This antibody detects both phosphorylated Tyr701 of p91 STAT1α and also the p84 splice variant STAT1β (referring to the company [cell signaling technology, MA, US] data sheet and references (#1 and #2). Additionally, the company has performed CIP (Calf intestinal alkaline phosphatase) membrane treatment using this antibody and has shown that both signal detected by $pSTAT1^{Tyr701}$ antibody was completely knocked down upon phosphatase membrane treatment with each lot of this antibody. Consistently, our data here show both clear bands and both of which were EGFR signalling dependent as expected. Throughout this thesis, both bands (pSTAT1α and pSTAT1β) were quantified for pSTAT1.)

#1. Zahoor MA, Xue G, Sato H, Murakami T, Takeshima SN, Aida Y. HIV-1 Vpr induces interferon-stimulated genes in human monocyte-derived macrophages. PLoS One 2014;9(8):e106418.

#2. Xu W, Edwards MR, Borek DM, Feagins AR, Mittal A, Alinger JB, et al. Ebola virus VP24 targets a unique NLS binding site on karyopherin alpha 5 to selectively compete with nuclear import of phosphorylated STAT1. Cell host & microbe 2014;16(2):187-200.

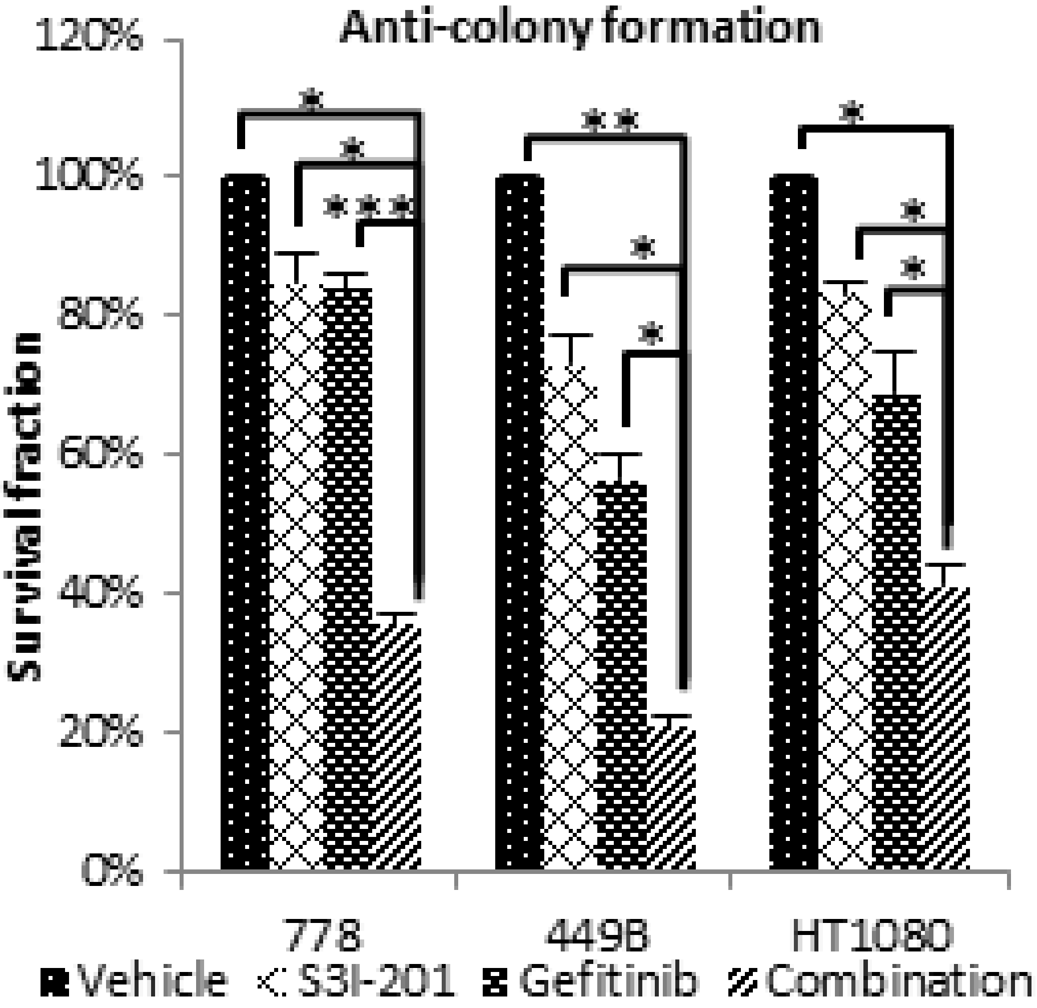

Supplementary Figure S3: Sarcoma cell lines 778, HT1080 and 449B achieved synergistic anti-colony formation after combination treatment with gefitinib and S3I-201. Clonogenic assay in STS cell lines treated with gefitinib (10 µM), S3I-201 (5 µM) or combination of both drugs. The survival fraction (from duplicate experiments, shown in Figure 3D) was calculated by dividing the number of colonies from treated cells by the number of colonies from untreated cells. The percentage of untreated cells was considered as 100%. * $p < 0.05$, ** $p < 0.01$, *** $p < 0.005$

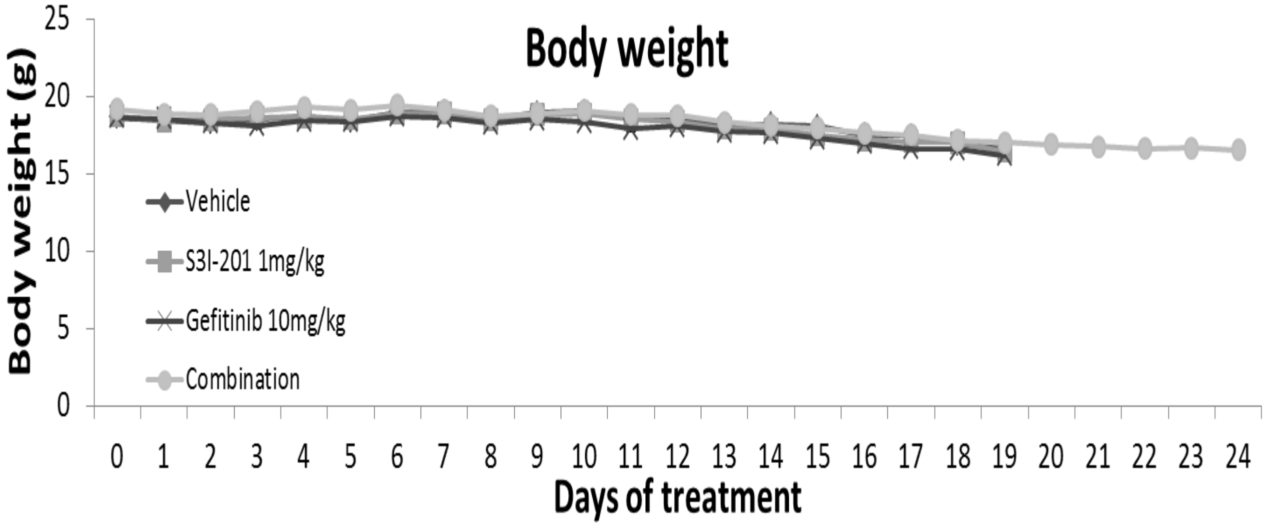

Supplementary Figure S4: Mouse body weights were measured daily and no difference between different groups were found. All p values > 0.05

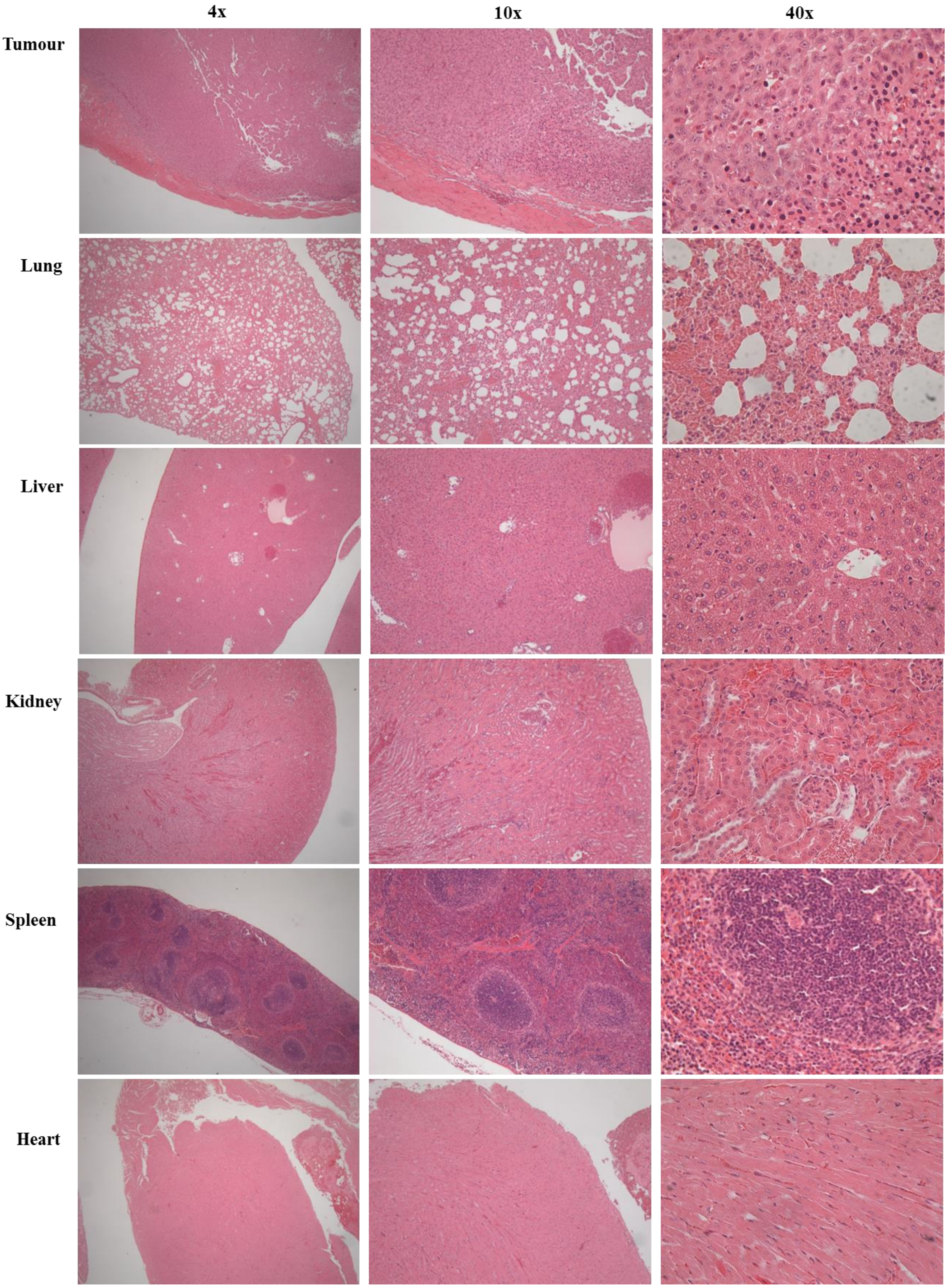
4x
10x
40x
Tumour
Lung
Liver
Kidney
Spleen
Heart

Supplementary Figure S5: Histochemical analysis of the tumours and organs (lung, liver, kidney, spleen and heart) harvested from the sacrificed mice with orthotopic human fibrosarcoma xenografts. HE staining shows neither metastatic tumours nor histological toxicities in all organs.

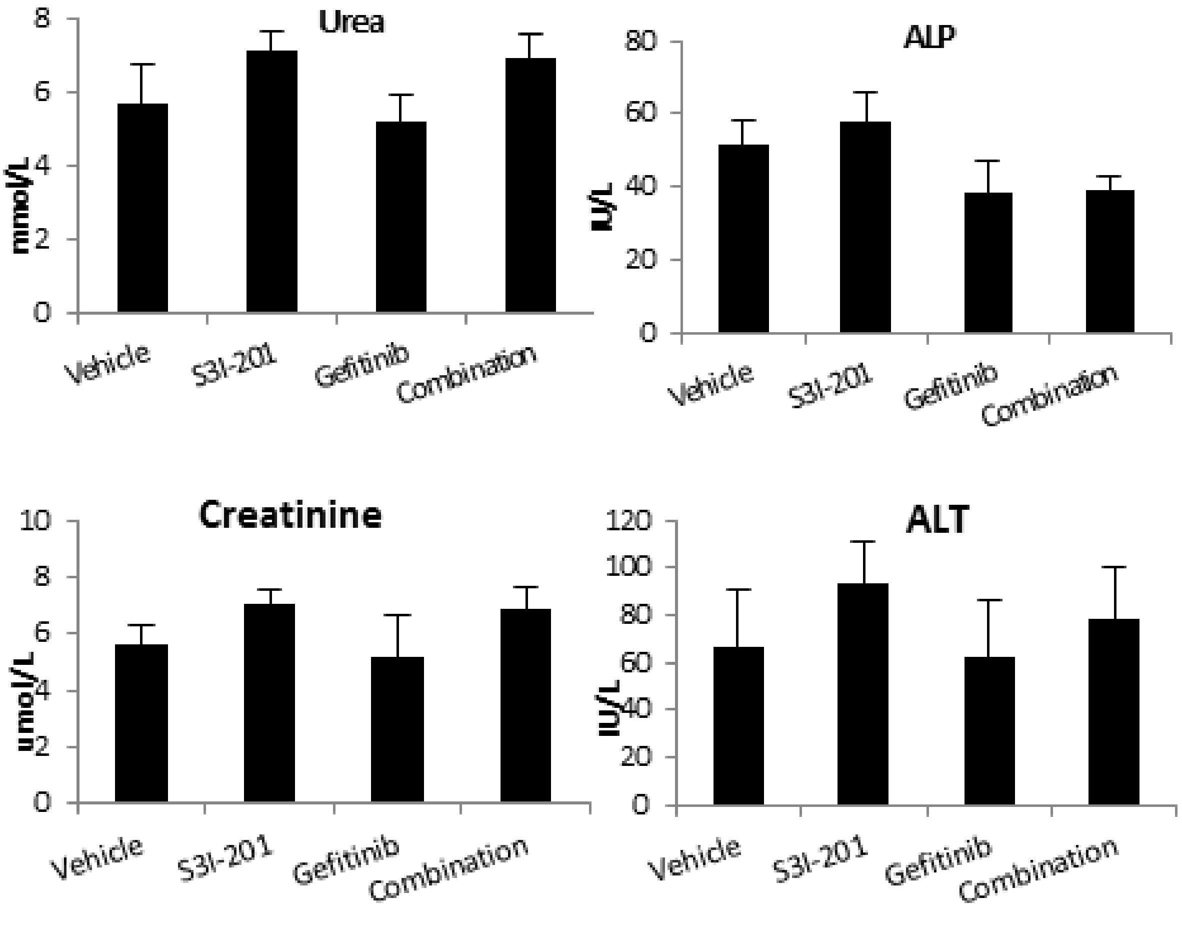

Supplementary Figure S6: Toxicity analysis of serum biomarkers after administration of S3I-201 and gefitinib alone or in combination. Quantitative data show means ± standard error of the mean (SEM). All *p* values (compared to vehicle control) >0.05
